# Supplementary material for: Niche and Geographic Drivers Shape the Diversity and Composition of Endophytic Bacteria in Salt-Tolerant Peanut
Source: Microorganisms. 2025 Sep 26;13(10):2264. doi: 10.3390/microorganisms13102264 (PMC12566507; doi:10.3390/microorganisms13102264)
Supplement: Supplementary file 1 [file microorganisms-13-02264-s001.zip › microorganisms-3881975-supplementary.pdf]

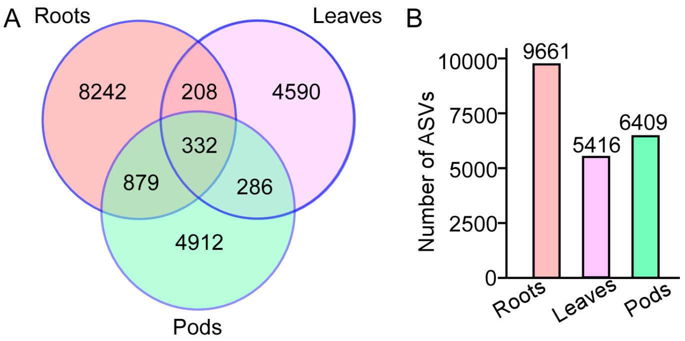

Figure S1. The Venn diagram of ASVs in the samples. The numbers of shared and unique ASVs in three organs are shown, including roots, leaves, and pods.

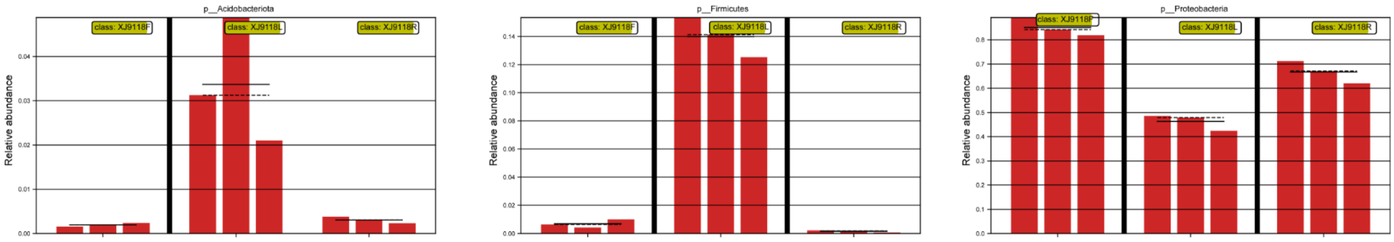

Figure S2. The relative abundance of endophytic bacterial phyla level in different tissues of XJ9118.

Table S1. The soil composition of the sampling sites—three different saline-alkali lands.

Collection Site	SO_4^{2-} (g/kg)	Cl^- (g/kg)	HCO_3^- (mg/kg)	pH	SOM (g/kg)	HN (mg/kg)	AP (mg/kg)	AK (mg/kg)	Salt (%)
Yili (XJ)	1.78	0.22	90.5	7.82	14.9	43.2	48.9	228	7.62
Baicheng (JL)	0.07	0.042	99.1	9.24	20	63.1	15.8	103	0.98
Dongying (DY)	0.28	0.36	8.31	7.64	14.4	58.2	32	338	2.76
